# LiDAR-Based Snowfall Level Classification for Safe Autonomous Driving in Terrestrial, Maritime, and Aerial Environments

**DOI:** 10.3390/s24175587

**Published:** 2024-08-28

**Authors:** Ji-il Park, Seunghyeon Jo, Hyung-Tae Seo, Jihyuk Park

**Affiliations:** 1 National Defense AI Center, Agency for Defense Development (ADD), 160, Bugyuseong-daero 488beon-gil, Yuseong-gu, Daejeon 34060, Republic of Korea; tinz64@kaist.ac.kr; 2 AUTOCRYPT GmbH, Salvatorplatz, 80333 München, Germany; coolwind@hotmail.co.kr; 3 Department of Mechanical Engineering, College of Creative Engineering, Kyonggi University, 154-42, Gwanggyosan-ro, Suwon 16227, Republic of Korea; 4 Department of Automotive Engineering, College of Digital Convergence, Yeungnam University, 280 Daehak-ro, Gyeongsan 38541, Republic of Korea

**Keywords:** snowfall level, LIOR filter, LiDAR point cloud, extreme weather

## Abstract

Studies on autonomous driving have started to focus on snowy environments, and studies to acquire data and remove noise and pixels caused by snowfall in such environments are in progress. However, research to determine the necessary weather information for the control of unmanned platforms by sensing the degree of snowfall in real time has not yet been conducted. Therefore, in this study, we attempted to determine snowfall information for autonomous driving control in snowy weather conditions. To this end, snowfall data were acquired by LiDAR sensors in various snowy areas in South Korea, Sweden, and Denmark. Snow, which was extracted using a snow removal filter (the LIOR filter that we previously developed), was newly classified and defined based on the extracted number of snow particles, the actual snowfall total, and the weather forecast at the time. Finally, we developed an algorithm that extracts only snow in real time and then provides snowfall information to an autonomous driving system. This algorithm is expected to have a similar effect to that of actual controllers in promoting driving safety in real-time weather conditions.

## 1. Introduction

When a person drives a car, driving under snowy conditions is bound to be different from driving on a clear day. This is because people drive safely with an instinctive intuition due to limited vision, slippery roads, and short braking distances when it snows. Therefore, to implement fully autonomous driving on the ground, it is essential to recognize the weather environment in the same way as humans and drive safely based on it because the driving pattern varies depending on the weather [1].

However, because the currently developed unmanned vehicles, ships, drones, etc., cannot check real-time weather conditions as humans can, such safe autonomous driving control is virtually impossible. Currently, self-driving research is mainly focused on sunny weather, and even related technological development is limited.

With self-driving vehicles, the most representative platform among self-driving platforms, as an example, the autonomous driving levels can be divided into six classes, and the current level is level 3 or 4. Some companies, such as Hyundai, Waymo, and Cruise, claim that they have achieved level 4 autonomous driving [2,3,4]. However, this achievement is questionable because the current technology has only been applied in some limited environments. Regardless, level 4 autonomous driving is clearly on the verge of completion and will be achieved soon.

According to the SAE (Society of Automotive Engineers), level 5 autonomous driving is nearly the same as level 4; the only difference is that in level 5, autonomous driving is possible everywhere in all conditions [5]. This designation includes extreme environments, a typical example of which is a snowfall environment. Accordingly, many companies that are about to achieve level 4 autonomous driving technology are conducting research on autonomous driving in snowy environments to reach level 5. Almost all automakers realize that the future of autonomous driving must extend beyond ideal conditions. The need for these studies is supported by the fact that more than half of the world’s regions receive snow, and 24% of weather-related crashes in the United States occur on snowy pavement [6].

Therefore, this study aims to develop a real-time snowfall recognition system that is essential for autonomous driving and present a new criterion for safe vehicle control. In particular, using the proposed real-time snowfall recognition information as input data in vehicle control will enable more stable acceleration, deceleration, and steering control, reducing unstable control such as slipping.

### 1.1. Related Research

Research on classifying and estimating weather using LiDAR sensors has been conducted through various methodologies. Initial efforts have focused on detecting atmospheric phenomena such as wind, dust storms, vortices, and atmospheric turbulence using atmospheric LiDAR sensors for weather observation [7,8,9].

Conversely, various studies have aimed to classify and estimate different types of weather using LiDAR sensors employed in autonomous vehicles, as examined in this paper. Dannheim conducted a study utilizing a fusion of LiDAR sensors and cameras to distinguish between snow, rain, and fog. To obtain accurate weather object information, the LiDAR reflections were processed using a Kalman filter for particle filtering [10]. Rivero installed a LiDAR sensor at a fixed location for nine months, collecting data by directing the sensor at an asphalt surface. By analyzing variations in background radiation and the number of points scanned, he developed an algorithm to distinguish between clear, foggy, rainy, and snowy weather conditions [11]. González-Jorg utilized mobile LiDAR data in conjunction with climate information from weather stations positioned along the road to evaluate driver visibility [12]. Tian analyzed the characteristics of roadside LiDAR data under snowy, rainy, and windy conditions, determining offset thresholds and developing an automated method to identify data corresponding to different weather conditions. In snowy or rainy environments, object detection distances are affected by noise, and this offset in detection distance is utilized to identify such conditions. Additionally, in windy environments, the vibration differences between ground points and objects shaken by the wind are used to detect the effects of wind [13]. Finally, Silva investigated weather detection using deep learning. He proposed a method employing a convolutional neural network to detect weather conditions by projecting LiDAR point cloud data into a 2D bird’s-eye view [14].

Weather detection using cameras has significantly advanced alongside developments in image processing and machine vision [15,16,17]. Early research employed traditional methods such as edge detection, contrast, and gradients to identify weather conditions. However, recent advancements have leveraged AI technologies to extract quantitative weather information from images. Notably, methods for detecting rain and fog using single-lens cameras have been developed [18], but this approach is limited to specific scenarios and struggles with integrating diverse weather conditions due to lengthy processing times.

Research focusing on weather and road condition assessment using in-vehicle camera systems has been active, and recent developments have introduced AI-based classification algorithms capable of detecting multiple weather conditions simultaneously. For instance, methods utilizing AdaBoost classifiers [19], as well as algorithms like RNN, LSTM, and CNN, have achieved high accuracy [20,21]. Additionally, studies employing fixed cameras, such as those installed in road weather information systems (RWISs) or CCTV, have also been conducted [22,23].

Moreover, to improve the reliability of data labeling, camera-based datasets for road surface estimation under various weather conditions have been proposed [24,25,26]. Consequently, there is an increasing need for novel methodologies and databases that can integrate diverse weather conditions comprehensively, highlighting the ongoing demand for effective weather detection research.

In addition to the widely used LiDAR and camera sensors, radar is also employed in autonomous vehicles for environmental sensing. Although radar is highly robust and capable of reliably perceiving the surrounding environment in extreme conditions such as snow or rain, this very robustness poses challenges for research focused on recognizing and differentiating specific weather conditions. Radar’s limited resolution in detecting precipitation and fog further limits its suitability for identifying particular weather phenomena. As a result, LiDAR and camera sensors are more commonly utilized for weather detection.

### 1.2. Research Necessity and Solution

Perception, one of the components of autonomous driving, is divided into detection and localization, and detection is further divided into environmental detection, such as traffic signs, traffic lights, and drivable space; and object detection [27]. Localization and object detection studies have been conducted in snowy environments, as described above [28,29,30], but research on environmental detection in snowy weather conditions is limited. This problem is caused by interpreting the environment only from the topographical aspect.

However, the environment includes meteorological conditions in addition to terrain conditions and features such as mountains, waves, and buildings, and people consider the weather as well as the terrain, so weather recognition is essential. In fact, this weather information is used in control to enable safe driving. Accordingly, we studied the perception of meteorological conditions, especially snowfall, rather than the recognition of objects and the surrounding terrain in a snowy environment, which has already been studied.

To this end, developing a method to check the amount of snowfall in real time in a snowfall environment is necessary. If possible, the use of autonomous driving sensors alone, without the use of additional sensors, is desirable. Of course, there is a way to install a meteorological sensor typically found in a weather station with high accuracy, but there is a limit to additional installation in terms of cost and power because many electronic devices are already attached to the vehicle. Additionally, when the vehicle is stationary or moving, the number of snow points acquired through LiDAR is nearly identical. This is due to the fact that even if the vehicle moves or the snow is quickly scattered by the wind, the speed of light from the LiDAR sensor beam is so high that it accurately captures the moment when the snow is located. For moving objects, snow accumulation on the LiDAR sensor surface could be addressed through hardware solutions, such as using a wiper, air blower, or protective coverings. Therefore, this issue was not considered in our study.

Representative self-driving sensors used for perception include LiDAR, radar, and camera systems. Radar uses an electromagnetic signal, so it cannot be easily used to measure snowfall because snow particles cannot be sensed. Of course, measuring snowfall on the surface using high-resolution space-borne radars is possible [31], but this study is about the classification of snowfall for falling snow, not snow accumulated on the surface, and radar sensors are not suitable for research purposes. High-resolution cameras can measure snow, but measuring the exact amount of snow located in a certain 3D voxel is difficult because it is measured using a 2D image. Of course, 3D data can be acquired with stereo cameras, but stereo vision is exceptionally dense and noisy, so real-time measurements are characterized by low position accuracy and a low computational speed.

LiDAR sensors can accurately measure the location of an object through point cloud data; therefore, they can be used to measure the amount of snowfall in real time. This requires a technology that can extract noise points generated by snow from LiDAR point cloud data, and accordingly, an intensity-based low-intensity outlier removal (LIOR) filter, which is fast and accurate, was used in this study [32]. To this end, we developed a real-time snow extraction algorithm based on an LIOR filter, obtained snowfall data from various snowy areas, and analyzed the number of snow particles extracted to classify and define the snowfall level. The detailed method is described in Section 2.

This paper is organized as follows: Section 2 describes the experimental method, including the snow extraction algorithm, hardware design, data acquisition, etc.; Section 3 summarizes the results of the snow extraction analysis and the newly defined snowfall level; and Section 4 concludes the paper. This is a new concept that can sense the amount of snowfall through the LiDAR sensor, which provides snowfall information about the local area where the actual vehicle is located, so it is expected to enable safe driving when used as input data for vehicle control such as acceleration, deceleration, and steering.

## 2. Experimental Method

### 2.1. Snow Extract Algorithm

Snow particles in a LiDAR point cloud can be extracted by reversely applying an existing LiDAR point cloud noise removal algorithm. Several algorithms are available for removing the noise points generated by snow, including a deep learning method [33], a distance-based noise removal method [34], and an intensity-based noise removal method [32,35]. Because the system proposed in this study needs to accurately extract only snow in real time, we decided to extract snow using an intensity-based LIOR filter with both high speed and accuracy. As shown in Table 1, the performance of the intensity-based LIOR was better than that of the distance-based DROR. This shows the same performance when the vehicle is stationary or moving because, as mentioned above, all snow particles can be scanned at once because the speed of the LiDAR beam is very high. This applies equally to both the distance-based method and the intensity-based method.

The accuracy of the LIOR filter in snow noise filtering is excellent. In particular, this filter yields high accuracy for true positives, which is the ratio of the number of filtered snow points to the number of real snow points. In addition, good performance is observed in noise removal, as the filter has a low false positive rate, which is the ratio of the number of filtered snow points compared with the number of non-snow points. In particular, the true positive rate, which is the ratio of the number of filtered snow points to the number of real snow points, indicates the accuracy of snow extraction, and the false positive rate, which is the ratio of the number of filtered snow points to the number of non-snow points, is close to 0; therefore, the filtering accuracy of this method is very high [32].

Because the LIOR filter is an intensity-based snow removal filter, a threshold is needed as a criterion for the extraction of snow points. The proposed system uses Ouster’s OS-1 LiDAR, which was used in the research that led to the development of the existing LIOR filter; therefore, the value shown in Equation (1) is set as the threshold, and Equation (2) describes the OS-1 LiDAR intensity curve required to calculate $I_{th}$. The difference from the existing LIOR filter is that the LIOR-based snow extraction filter does not simply remove snow but utilizes the removed snow point information, which is checked in real time and used to recognize the level of snowfall. Here, the variables $I_{th}$, AR, and $R_{s}$ represent the intensity threshold for snow point extraction, the area ratio, and the snow reflectance, respectively.

(1) $$I_{th}=I_{c}\cdot AR\cdot cos\left(\alpha \right)\cdot R_{s}=0.0469I_{c}.$$

(2) $$I_{c}=I_{ref}\cdot \frac{{D_{ref}}^{2}}{{D_{c}}^{2}}\cdot cos\left(\alpha \right)=4180\cdot \frac{5.5^{2}}{{D_{c}}^{2}}$$

Because the area of a snow particle is smaller than the size of the sensor beam and because the intensity of a snow particle varies depending on its reflectivity, the area ratio of the beam area to the snow area, the OS-1 LiDAR wavelength, and the snow particle size must be considered to calculate the exact intensity of the snow particles. Details of the parameters and constants in Equations (1) and (2) are described in the paper introducing the LIOR filter [32].

**Figure 1:**
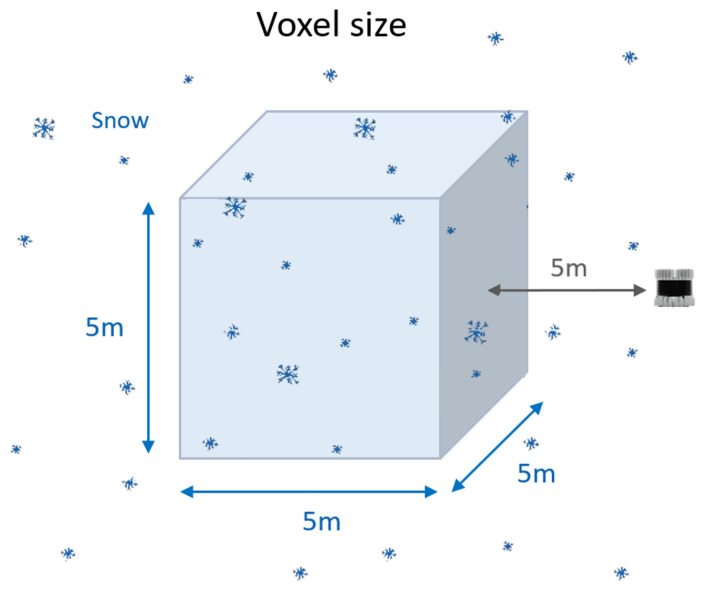
Voxel size for extracting snow points. Snow particle extraction was performed on a single 5 × 5 × 5 m voxel.

The overall flow of the LIOR-based snow extraction filter is described in the pseudocode in Algorithm 1. First, it is necessary to set the voxel size for extracting a snow point because the only drawback of a LIOR filter is that when filtering an entire point cloud, the processing speed is slow [32]. Therefore, various tests are conducted to identify an area in which real-time snow extraction is possible. If the voxel size is too large, the processing efficiency decreases due to unnecessary calculations, and if the voxel size is too small, the reliability of the number of snow points decreases; therefore, various tests are conducted to determine the voxel size to quickly and accurately extract snow. Accordingly, the voxel size is finally designated at 5 × 5 × 5 m, and the vertical field of view of the LiDAR is 45°. The voxel is positioned 5 m in front of the LiDAR so that all parts within the voxel can be sensed without adjustment (Figure 1).

In addition, we tried to extract snow points through DROR, a representative distance-based algorithm, but the extraction speed was slower than that of LIOR and the problem of not extracting all snow occurred, so LIOR was finally selected as a snow extractor; the related results are shown in Table 1. Also, as a result of testing the voxel size in four cases: 1 × 1 m, 3 × 3 × 3 m, 5 × 5 × 5 m, and 7 × 7 × 7 m, we selected 5 × 5 × 5 m as the final voxel size, where snow extraction is possible in real time and standard deviation changes in data values begin to stabilize, as shown in Table 2. The location of the voxel is selected as an area where the object does not normally exist, but even if the object comes inside the voxel area, it can be converted into normalized data and then classified for snowfall.
**Algorithm 1** Snow extraction pseudocode**for** p∈P **do**    // Define voxel size to extract snow points    Set the voxel size to 5×5×5 m    // Extract snow points based on an intensity threshold    **if** $I_{p}\leq I_{\mathrm{thr}}$ **then**        point *p* classified as a snow point $p_{s}$    **else**        point *p* classified as an outlier $p_{\mathrm{out}}$        // Extract snow points again from outliers        Count the number of neighbors $n_{p}$        **if** $n_{p}\leq n_{\mathrm{thr}}$ **then**           outlier $p_{\mathrm{out}}$ reclassified as snow $p_{s}$        **end if**    **end if****end for**

### 2.2. Hardware Design

**Figure 2:**
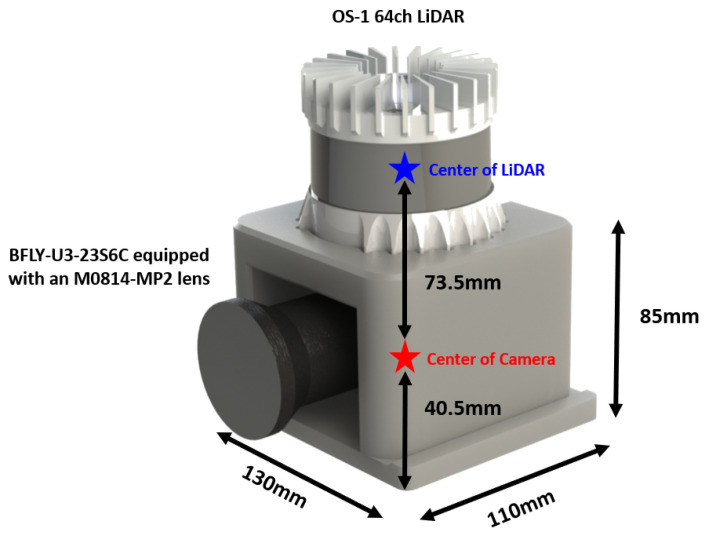
Design of the sensor mounting system.

The LiDAR sensor used for snow point extraction was an OS-1 64ch LiDAR (Ouster Inc., San Francisco, CA, USA), and the rotation rate, vertical resolution, and horizontal resolution were set to 10 Hz, 64 ch, and 1024 pts, respectively. Accordingly, a total of 65,536 points were obtained per scan. In addition, given the difficulty of accurately checking the weather using only a LiDAR sensor at the time when the snow data were acquired, a vision camera was used to check the weather. A Blackfly BFLY-U3-23S6C (Teledyne FLIR, Wilsonville, OR, USA) equipped with an M0814-MP2 (Teledyne FLIR, Wilsonville, OR, USA) lens was used for the camera, and the resolution and FPS were set to 1024 × 720 and 30, respectively (Figure 2). The camera was not installed to fuse with the LiDAR but to check peculiarities in the weather (changes that are difficult to identify with LiDAR, such as snow, rain, or hail) or noise caused by unknown objects such as leaves and plastic bags. Finally, snow datasets were obtained by connecting the LiDAR and camera to a laptop using the Robot Operating System (ROS1) middleware program. The laptop computing hardware was an Intel(R) Core(TM) i7-10870H CPU @ 2.20 GHz with 32 GB of RAM.

### 2.3. Data Acquisition

The experimental data were obtained from snowy northern regions in South Korea, Sweden, and Denmark. In South Korea, snow data were acquired in the Gangwon-do region for one month. In Sweden and Denmark, snow data were acquired for two weeks in Luleå and Copenhagen, respectively. For the experimental areas in each country, a location where sufficient space could be secured for the voxel size defined above was selected.

**Figure 3:**
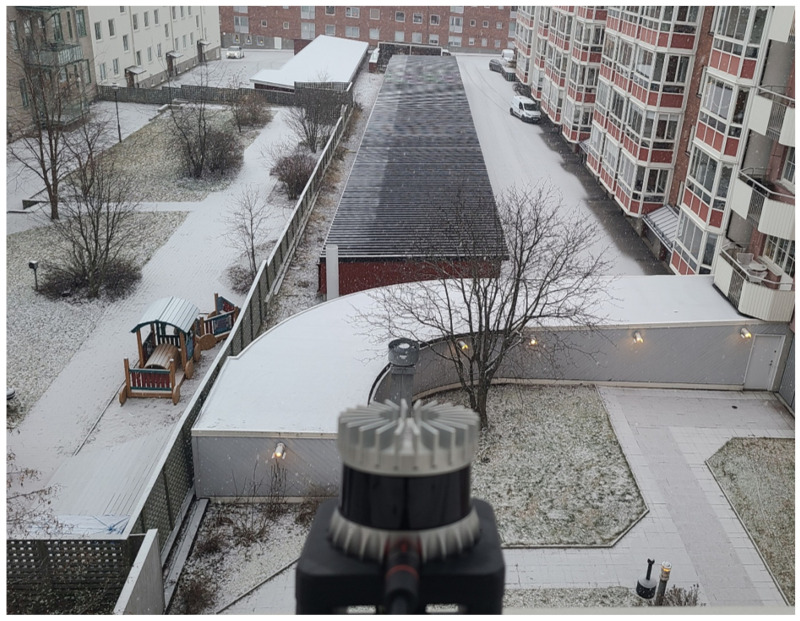
Experimental environment for snow data collection (Sweden). The LiDAR sensor was installed to be exposed to the outdoors and acquired both frontal and lateral outdoor data.

**Figure 4:**
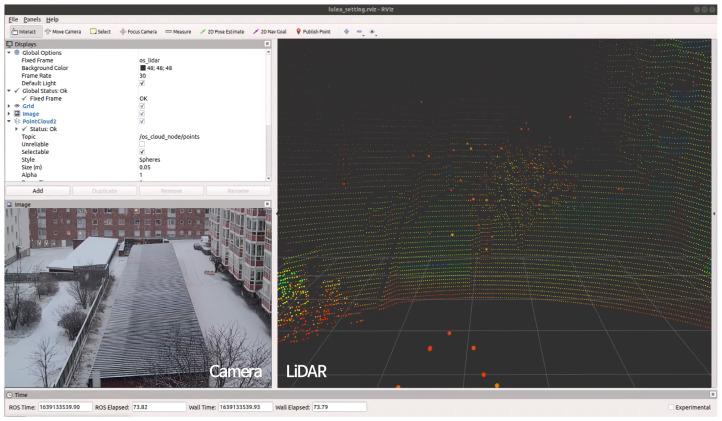
Sample data visualized through the ROS RViz. The camera image was used to visually check the noise caused by unidentified objects, such as leaves, plastic bags, and other garbage, not snow.

Figure 3 shows the location where snow data were acquired in Luleå, Sweden, and Figure 4 shows a sample of the acquired data. The data were collected from the 4th floor of a building with no objects within 120 m in front of the effective detection distance of the LiDAR sensor for snow extraction. The reason why the voxel location was selected as an object-free area on the 4th floor was to ensure that only snow points would be extracted in this area. If there is an object in a given area, the number of snow points sensed will decrease in proportion to the volume of the object; therefore, to extract the exact number of snow points within the designated voxel size, it is necessary to select an area without any objects present as a default. In particular, the data collected by drones driving in the air or ships driving at sea are often ideal because it is very likely that there are no objects in the voxel area. Initially, it was expected that there would be many variations in measuring the number of snow particles caused by the wind in such an open space, especially in the voxel area in the air. However, even in an environment where various wind directions and wind speeds change randomly during an actual snowfall measurement experiment, the speed of the wind was not a problem at all in measuring the number of snow points because the light speed of the LiDAR beam was extremely fast. Similar to the data acquired in Luleå, in South Korea and Denmark, snow data were obtained at locations with sufficient voxel spaces for snow extraction.

**Figure 5:**
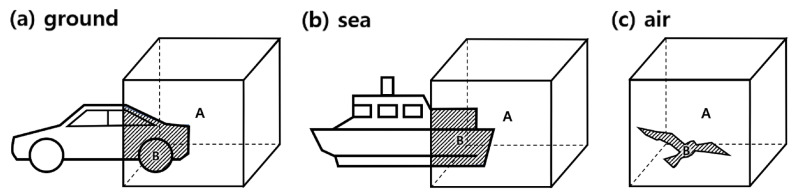
Various cases in which an object may exist within a set voxel.

However, as shown in Figure 5, for vehicles driving on the ground, there is a high possibility that an object will exist within the voxel area, and although it is less likely than on the ground, for drones or ships traveling in the air and at sea, objects such as other ships or birds may also exist within the voxel area. In this case, as shown in Equation (3), the normalized value can be obtained by measuring the number of snow points present in area (A−B) and then dividing it by the volume of (A−B). Here, the variables $P_{snow(A-B)}$, $V_{\left(A-B\right)}$, and $P_{norm(A-B)}$ represent the number of snow points acquired from the volume excluding object B from voxel A (5 × 5 × 5 m), the snow volume excluding object B from voxel A, and the values normalized to a 1 × 1 × 1 m volume, respectively. To put it simply, it means that the number of snow in the voxel area is divided by the voxel volume for the area where the snow is detected and finally calculates the number of normalized snow for a volume of 1 × 1 × 1 m.

(3) $$P_{norm(A-B)}=\frac{P_{snow(A-B)}}{V_{\left(A-B\right)}}$$

As such, when the proposed algorithm is applied to actual systems such as unmanned vehicles, drones, and ships, if an object exists in the voxel, as in Figure 5, the total number of snow points $P_{\left(A\right)}$ considering the area obscured by the object can be extracted by multiplying the normalized value by the volume of A, as shown in Equation (4). This equation is applied for each LiDAR scan for real-time analysis.

(4) $$P_{\left(A\right)}=P_{norm(A-B)}\cdot V_{\left(A\right)}$$

Data were acquired day and night, and the amount of snowfall predicted in the weather forecast and the amount of actual snowfall were regularly checked and recorded. The snow data were acquired using LiDAR as the main sensor, and a mono camera was only used to visually check the daytime and nighttime conditions and the surrounding snowy environment. The acquired raw data were edited and stored every 3 min during times of heavy snowfall, and a total of 1800 scans were obtained per sample file because the rotation rate of the LiDAR was set to 10 Hz.

## 3. Result

### 3.1. Analysis of Snow Extraction

First, the number of snow particles was extracted from each sample of point cloud data through an LIOR-based snow extraction filter. Among the snow data acquired in each country, four samples with various characteristics were selected within a given range so that the results did not significantly overlap. The extracted snow information included the number of extracted snow points in each frame; the X, Y, and Z coordinates of points; and the intensity values of the extracted points. Based on this information, the mean and standard deviation of the snow points for each frame extracted for each sample of data were calculated.

**Figure 6:**
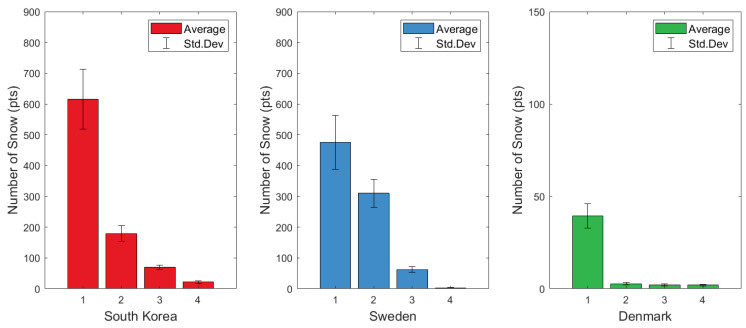
The result of extracting snow from four representative data points obtained in each country. The numbers 1 to 4 at the bottom refer to the order in which they are sorted according to the amount of snowfall. The number of snow particles extracted from the data obtained in Denmark was very small compared with the numbers in South Korea and Sweden, so the *y*-axis range was changed to 100.

Figure 6 shows the results of snow extraction from the four data samples acquired in South Korea, Sweden, and Denmark. As the average number of extracted snow particles increases, the standard deviation also increases, and accurate values for each sample are shown in detail in Table 3. Snow data collected from South Korea and Sweden were not only gathered during ordinary snow events but also on days with heavy snow advisories and warnings. This resulted in snow records where the number of snow particles extracted from the 5 × 5 × 5 m voxel exceeded 400 per frame. Additionally, dry snow records were obtained because it snowed at a very low temperature of −10° [36].

The snow data from Denmark indicated relatively small amounts of snow in December because it did not snow as much as expected. In addition, in Denmark, the temperature was not very low and varied between 1° and −2°; thus, rain was common, the humidity was high, and records of wet snow with small particles were mainly obtained [32]. In addition, by analyzing the average intensity of the snow points, the correlation between the number of snow points and the intensity of snowfall was confirmed. As the number of extracted snow particles increases, the size of the snow particles also tends to increase, so the average intensity value tends to increase as well. However, interestingly, a comparison of the first and second datasets obtained in Sweden shows that this is not always the case. This is because the number of falling snow particles may be small even though the size of the snow particles is large.

**Figure 7:**
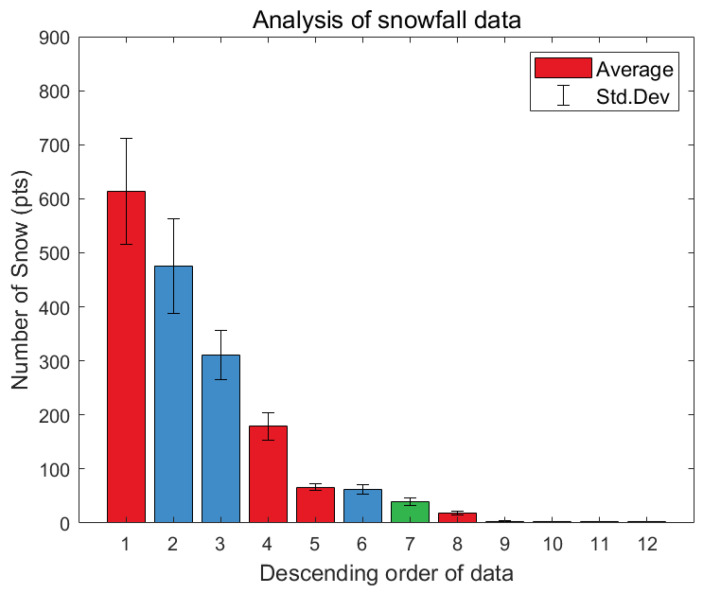
Descending order of extracted snow data. The 9th data point is from Sweden, and the 10th to 12th data points from Denmark. Red, blue, green, and boxes are data acquired from South Korea, Sweden, and Denmark, respectively.

Figure 7 shows all twelve data samples, with four data samples obtained from each country, in order from the largest to the smallest number of extracted snow particles. As a result of acquiring data from various countries and environments, meaningful results with various numbers of snow particles were obtained.

### 3.2. Defining the Snowfall Level

#### 3.2.1. Snowfall Forecast Standards by Country

To classify the snowfall level, the criteria to be used must first be determined. Various countries worldwide have distinct standards for snow forecasting. In the case of the United States, the winter weather service issues three types of forecasts: watches, advisories, and warnings. Additionally, there are three types of issued warnings, namely winter storm warnings, ice storm warnings, and blizzard warnings, and advisories are issued as winter weather advisories or freezing rain advisories [37]. Based on the criteria of this study, winter storm warnings and winter weather advisories were considered. A winter storm warning indicates that heavy snow of at least 6 inches in 12 h or at least 8 inches in 24 h is expected. It can also be issued if sleet accumulation is at least half an inch. A winter weather advisory is issued for one or more of the following cases: snowfall of 3 to 5 inches in 12 h, sleet accumulation up to a quarter inch, freezing rain in combination with sleet and/or snow, or blowing snow [37].

South Korea’s snowfall forecast is divided into a heavy snow advisory and a heavy snow warning. A heavy snow advisory is issued when more than 5 cm of new snow in 24 h is expected, and a heavy snow warning is issued when there is more than 20 cm of new snow in 24 h [38]. In Europe, yellow, orange, and red are used for weather warnings and are commonly issued for wind, rain, snow/ice, temperature, fog, and thunderstorms. A yellow warning is issued when the weather is potentially dangerous and the snowfall is less than 3 cm. Orange warnings are issued in dangerous weather when snow amounts between 3 and 8 cm have accumulated. A red warning is issued in very dangerous weather with more than 8 cm of snow [39].

The overall results are shown in Table 4, and although the figures vary slightly by country, all countries issue snowfall forecasts based on the cumulative snowfall. One difference is that Europe only considers snowfall and not the intensity. However, not all countries perform snow forecasts based on the intensity of snowfall in real time. Accordingly, this study proposes criteria for predicting real-time snowfall based on the existing snowfall forecast standards and the results of analyses using the acquired snowfall data.

#### 3.2.2. Classification by Snowfall Forecast

A snowfall level criterion through the snowfall forecast and the measurement of the number of snow particles through LiDAR was established. This is essential information to identify local snowfall information in the area where the vehicle is located, and it is a new criterion because it is the first method of classifying snowfall by considering the amount of snow measured through LiDAR and the weather forecast. This cannot be replaced by weather station information that predicts for a wide range of regions. The snow data obtained during a heavy snow advisory or a period of red snow warning were classified into the first group, the snow data obtained during a heavy snow warning or a period of orange snow warning were classified into the second group, and the data obtained on a day with no snow forecast or a yellow snow warning were classified into the third group.

**Figure 8:**
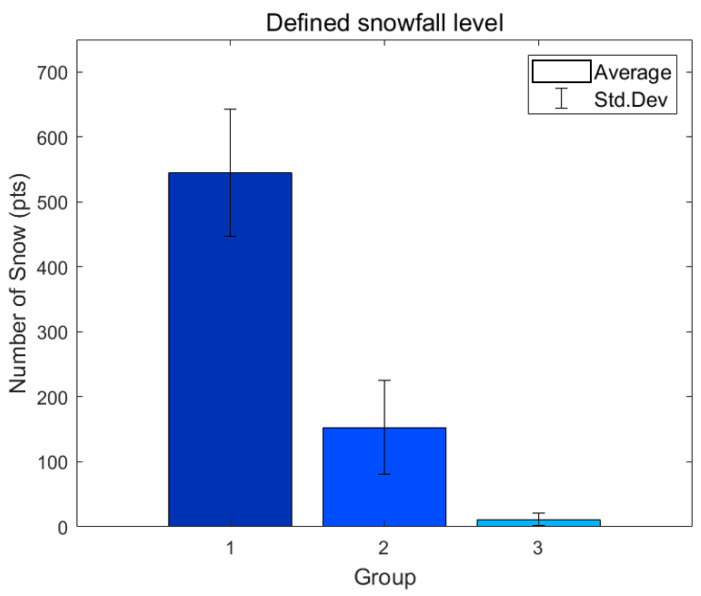
Snow analysis results for three groups classified based on weather forecasts.

**Figure 9:**
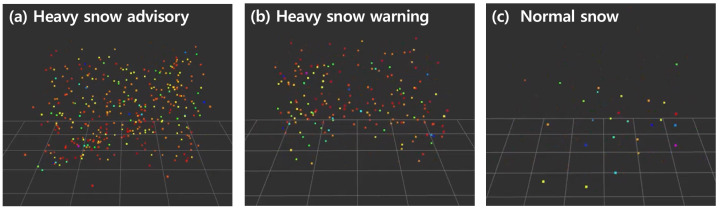
Result of snow point extraction by snowfall forecast.

Figure 8 and Table 5 are the results of calculating the mean and standard deviation of the number of snow particles for these three groups, and Figure 9 shows the result of snow particle extraction for these three groups. The results show that the number of snow particles for each forecast is clearly classified, and the actual measured number of snow particles matches the snowfall forecast. However, analyses were difficult because the data were divided into three groups according to the forecast, so the classification criteria for each group could not be easily set according to the number of snow particles. Additionally, the distribution of the data was not accurately expressed because the mean and standard deviation were calculated by dividing the data into only three groups, so values outside the standard deviation range were not intuitively considered. As such, the method of classifying snowfall at only three levels struggles to accurately classify the various snowfall environments in a local area where an actual autonomous vehicle travels due to the large gap in each stage of sensing, and further group subdivision is needed.

#### 3.2.3. Classification Based on Actual Snow Measurements

**Figure 10:**
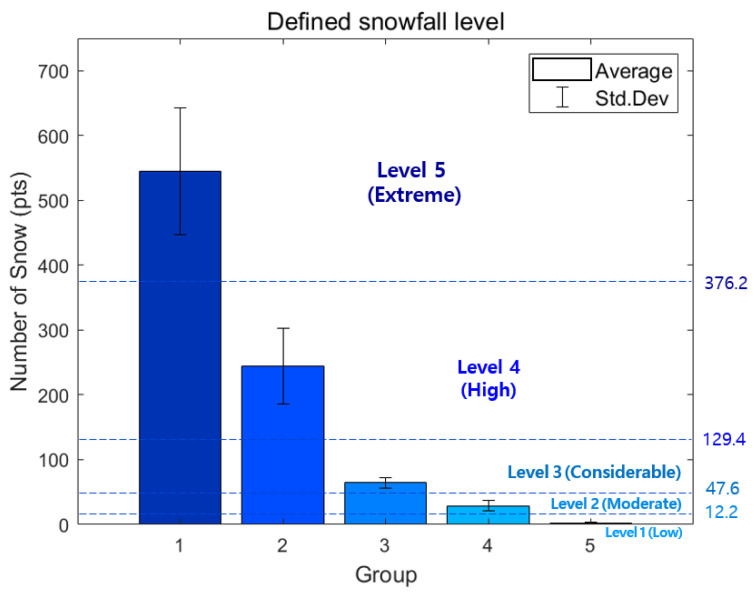
Defining the snowfall level for five groups.

Accordingly, groups 2 and 3, which contain various records, were further subdivided into two additional groups, and finally, the data were divided into a total of five groups. When divided into five groups based on actual acquisition data instead of three groups based on snowfall forecasts, more detailed snow particle numbers were derived, as shown in Figure 10 and Table 6, making it possible to accurately specify the range of the number of snow particles based on the snowfall level. This increase in accuracy is because the classification is based on real-time data acquired from an actual area instead of snowfall forecasts that are less accurate for local areas because they are issued over a wide range of regions and times. This means that our proposed LiDAR-base d snowfall level recognition algorithm shows higher accuracy than only considering snowfall forecasts when checking the exact snowfall level for the local area where the vehicle is located, and this accurate snowfall information enables safer vehicle control. On the other hand, these data in Table 6 also have some limitations, and for small-sized snow particles, there may be cases in which they are not returned depending on the reflectance, and in the case of snow located as overlapping the direction in which light travels, light does not reach and may not be counted as the number of snow particles. In addition, because the number of LiDAR channels increases, if the number of channels increases, our proposed snow level range increases in proportion to the number of channels. Nevertheless, our proposed criteria can be said to be a sufficiently meaningful result for classifying snowfall through the snowfall forecast and the number of snow measured by LiDAR.

**Figure 11:**
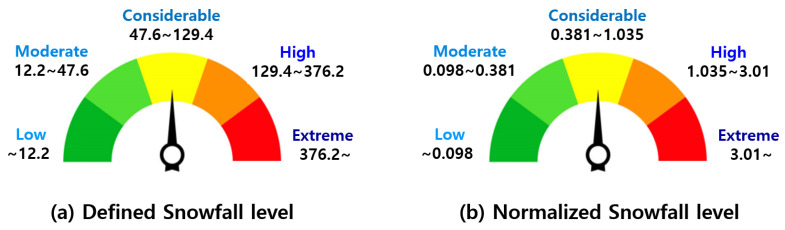
The final result of the snowfall level for five groups.

The upper range was calculated as the average of the value obtained by subtracting the standard deviation from the mean of the upper group and the value obtained by adding the standard deviation from the mean of the corresponding group. Similarly, the bottom range was calculated as the average of the value obtained by subtracting the standard deviation from the mean of the corresponding group and the value of adding the standard deviation from the mean of the lower group. Finally, five snowfall levels were defined: extreme, high, considerable, moderate, and low. The range of snow particle counts for each snowfall level is shown in Figure 11 and in Table 7. Figure 11b and range (b) in Table 7 are the data normalized to 1 × 1 × 1 m; these results can be applied to voxels of various sizes when implementing the system in practice.

One important point here is that the snowfall level range we proposed does not mean the total amount of actual snow in the voxel. A total of 65,536 points (vertical resolution 64 × horizontal resolution 1024) are sent for the 64-channel LiDAR sensor, but it cannot cover the entire area in the voxel because there are places where the beam does not reach. Therefore, the actual number of snow will be larger than the number we proposed. Additionally, as the proposed snow cover range is derived based on 64 channels, so it can be applied by multiplying by two when using a 128-channel LiDAR sensor and dividing by four when using a 16-channel LiDAR sensor.

There may be an opinion that it is inaccurate to classify the snowfall level based only on the average and standard deviation of totals extracted from limited data; however, it is very meaningful for the driver’s safe vehicle control because the amount of snow and snowfall level information obtained through the LiDAR sensor can be used to predict the amount of snow accumulated on the ground, the braking distance, and the visible distance.

## 4. Conclusions

In this study, a system was proposed where snowfall levels were defined and classified using snow data obtained from LiDAR in various snowy countries. In the future, this system is expected to contribute to the implementation of fully autonomous driving at a level similar to that of humans because snowfall information, which is identified in real time while driving, helps support the stable control of autonomous driving platforms.

In future studies, various information, such as temperature, humidity, and wind speed, will be used to more accurately classify snowfall stages, and we will expand this study by collecting additional snow data in more extreme snow environments. In particular, considering these characteristics, a more accurate snowfall level classification system will be developed because temperature and humidity determine whether snow is wet or dry, which affects the size and amount of snow. In addition, we will develop a system that provides rainfall levels in addition to snowfall levels by distinguishing snow from rain based on the temperature.

## Figures and Tables

**Table 1:** Comparison of snow extraction performance. LIOR shows good performance in extracting snow in terms of computational efficiency and speed.

		DROR	LIOR	Ground Truth
Accuracy	True Positive Rate (%)	93.72	99.87	100
	False Positive Rate (%)	25.13	0.07	-
Speed	Frame Per Second (FPS)	2.12	10.0	10.0 Hz

**Table 2:** Comparison of std. dev. of the snow number and snow points extraction speed by voxel size. The 5 × 5 × 5 m size satisfies both the snow data reliability and extraction speed.

	Size of Voxel			
	1 × 1 × 1 m	3 × 3 × 3 m	5 × 5 × 5 m	7 × 7 × 7 m
Std. dev. of noise points	5.24	3.72	2.42	2.35
Speed (FPS)	10.0	10.0	10	9.31

**Table 3:** Result of the snow data acquisition.

	Order	Average	Std. Dev.	Intensity (Average)	Snowfall		Snowfall Warning (Forecast)
					Forecast	Actual	
South Korea	1	615.8	98.37	125.2	6.0 cm	6.2 cm	Heavy snow advisory
	4	179.7	26.18	86.2	2.0 cm	1.8 cm	Heavy snow warning
	5	66.52	7.32	63.3	1.0 cm	1.0 cm	Heavy snow warning
	8	18.91	4.19	24.7	0.5 cm	0.7 cm	Not heavy snow
Sweden	2	477.1	88.74	94.8	5.0 cm	5.0 cm	Heavy snow advisory
	3	309.8	45.85	98.3	3.0 cm	3.1 cm	Heavy snow warning
	6	64.11	8.98	67.7	1.0 cm	1.0 cm	Heavy snow warning
	9	3.57	0.72	15.2	0∼0.1 cm	0.1 cm	Not heavy snow
Denmark	7	39.78	6.81	7.1	0.5 cm	0.7 cm	Not heavy snow
	10	3.02	0.89	13.9	0∼0.1 cm	0 cm	Not heavy snow
	11	2.74	0.61	12.5	0∼0.1 cm	0 cm	Not heavy snow
	12	2.07	0.52	12.8	0∼0.1 cm	0 cm	Not heavy snow

**Table 4:** Standards for snowfall forecasts in each country.

		Standard
US	Advisory	More than 6 inches (15.24 cm) in 12H or 8 inches (20.32 cm) in 24H
	Warning	3∼5 inches (7.62∼12.7 cm) in 12H
South Korea	Advisory	More than 20 cm in 24H
	Warning	More than 5 cm in 24H
Europe	Red	More than 8 cm
	Orange	Between 3∼8 cm
	Yellow	Less than 3 cm

**Table 5:** Average and std. dev. of snow particle numbers for the three groups.

Snowfall Level	Data	Amount of Snow	
		Average	Std. Dev.
Heavy snow advisory (red)	Group 1	546.2	98.02
Heavy snow warning (orange)	Group 2	153.5	73.36
No forecast (yellow)	Group 3	11.7	9.68

**Table 6:** Average and std. dev. of snow particle numbers for five groups. Snowfall data were analyzed using data acquired for 8 weeks in South Korea and 4 weeks in Sweden and Denmark.

Snowfall Level	Data	Number of Snow Particles	
		Average	Std. Dev.
Heavy snow advisory	Group 1	546.2	98.02
Heavy snow warning	Group 2	245.1	59.11
	Group 3	64.9	7.86
No forecast	Group 4	29.7	8.43
	Group 5	2.35	0.75

**Table 7:** Definition of snowfall level. Range (a) is the value when the voxel size is 5 × 5 × 5 m, and range (b) is the normalized value to 1 × 1 × 1 m.

	Snowfall Level				
	Extreme	High	Considerable	Moderate	Low
Range (a)	376.2∼	129.4∼376.2	47.6∼129.4	12.2∼47.6	∼12.2
Range (b)	3.01∼	1.035∼3.01	0.381∼1.035	0.098∼0.381	∼0.098

## Data Availability

All data generated or analyzed during this study are included in this published article.

## Abbreviations

The following abbreviations are used in this manuscript:

LiDAR	Light detection And ranging
LIOR	Low-intensity outlier removal
WADS	Winter Adverse Driving dataSet
CADC	Canadian Adverse Driving Conditions Dataset

## References

[1] Kurup A., Bos J. (2023). Winter adverse driving dataset for autonomy in inclement winter weather. Opt. Eng..

[2] Hyundai (2022). Hyundai × Motional-Bringing IONIQ 5 Robotaxis to the Streets from 2023. https://www.hyundai.com/worldwide/en/brand/robotaxis.

[3] Waymo (2022). Safety Report and Whitepapers. https://waymo.com/safety.

[4] McEachern S. (2021). Cruise founder takes company’s first driverless ride on SF Streets: Video. GM Authority.

[5] SAE International (2018). SAE International Releases Updated Visual Chart for Its “Levels of Driving Automation” Standard for Self-Driving Vehicle.

[6] U.S. Department of Transportation Fed-Eral Highway Administration (2021). https://ops.fhwa.dot.gov/weather/weather_events/snow_ice.htm.

[7] Atlas D., Korb C.L. (1981). Weather and climate needs for lidar observations from space and concepts for their realization. Bull. Am. Meteorol. Soc..

[8] Belegante L., Talianu C., Nemuc A., Nicolae D. (2011). Detection of local weather events from multiwavelength lidar measurements during the EARLI09 campaign. Rom. J. Phys..

[9] Pentikäinen P., O’Connor E.J., Ortiz-Amezcua P. (2023). Evaluating wind profiles in a numerical weather prediction model with Doppler lidar. Geosci. Model Dev..

[10] Dannheim C., Icking C., Mäder M., Sallis P. Weather detection in vehicles by means of camera and LIDAR systems. Proceedings of the 2014 Sixth International Conference on Computational Intelligence, Communication Systems and Networks.

[11] Vargas Rivero J.R., Gerbich T., Teiluf V., Buschardt B., Chen J. (2020). Weather classification using an automotive lidar sensor based on detections on asphalt and atmosphere. Sensors.

[12] González-Jorge H., Díaz-Vilariño L., Lorenzo H., Arias P. (2016). Evaluation of driver visibility from mobile lidar data and weather conditions. Int. Arch. Photogramm. Remote Sens. Spat. Inf. Sci..

[13] Tian Y. (2021). Identification of Weather Conditions Related to Roadside LiDAR Data. Master’s Thesis.

[14] Da Silva M.P., Carneiro D., Fernandes J., Texeira L.F. MobileWeatherNet for LiDAR-Only Weather Estimation. Proceedings of the 2023 International Joint Conference on Neural Networks (IJCNN).

[15] Robert G., Hallowell P., Michael P., Matthews P., Pisano A. Automated extraction of weather variables from camera imagery. Proceedings of the 2005 Mid-Continent Transportation Research Symposium.

[16] Hautiere N., Tarel J.P., Lavenant J., Aubert D. (2006). Automatic fog detection and estimation of visibility distance through use of an onboard camera. Mach. Vis. Appl..

[17] Ozcan K., Sharma A., Knickerbocker S., Merickel J., Hawkins N., Rizzo M. (2020). Road weather condition estimation using fixed and mobile based cameras. Advances in Computer Vision: Proceedings of the 2019 Computer Vision Conference (CVC), Volume 1.

[18] Bossu J., Hautiere N., Tarel J.P. (2011). Rain or snow detection in image sequences through use of a histogram of orientation of streaks. Int. J. Comput. Vis..

[19] Yan X., Luo Y., Zheng X. (2009). Weather recognition based on images captured by vision system in vehicle. Advances in Neural Networks—ISNN 2009: 6th International Symposium on Neural Networks, ISNN 2009 Wuhan, China, May 26–29, 2009 Proceedings, Part III.

[20] Khan M.N., Ahmed M.M. (2020). Trajectory-level fog detection based on in-vehicle video camera with TensorFlow deep learning utilizing SHRP2 naturalistic driving data. Accid. Anal. Prev..

[21] Khan M.N., Ahmed M.M. (2022). Weather and surface condition detection based on road-side webcams: Application of pre-trained convolutional neural network. Int. J. Transp. Sci. Technol..

[22] Jonsson P. Classification of road conditions: From camera images and weather data. Proceedings of the 2011 IEEE International Conference on Computational Intelligence for Measurement Systems and Applications (CIMSA) Proceedings.

[23] Sirirattanapol C., Nagai M., Witayangkurn A., Pravinvongvuth S., Ekpanyapong M. (2019). Bangkok CCTV image through a road environment extraction system using multi-label convolutional neural network classification. ISPRS Int. J. Geo-Inf..

[24] Cordes K., Reinders C., Hindricks P., Lammers J., Rosenhahn B., Broszio H. Roadsaw: A large-scale dataset for camera-based road surface and wetness estimation. Proceedings of the IEEE/CVF Conference on Computer Vision and Pattern Recognition.

[25] Cordes K., Broszio H. Camera-Based Road Snow Coverage Estimation. Proceedings of the IEEE/CVF International Conference on Computer Vision.

[26] Goberville N.A., Prins K.R., Kadav P., Walker C.L., Siems-Anderson A.R., Asher Z.D. (2023). Snow coverage estimation using camera data for automated driving applications. Transp. Res. Interdiscip. Perspect..

[27] Udacity (2022). Udacity’s Self Driving Nano Degree Program. https://www.udacity.com/course/self-driving-car-engineer-nanodegree-nd0013.

[28] Ford Media Center (2016). Ford Conducts Industry-First Snow Tests of Autonomous Vehicles–Further Accelerating Development Program.

[29] Torc (2018). Torc Self-Driving Car Dashes through Snow.

[30] Sensible4 (2022). Unique Technology for All-Weather Self-Driving Vehicles. https://sensible4.fi/technology/.

[31] Liu G. (2020). Radar snowfall measurement. Satellite Precipitation Measurement: Volume 1.

[32] Park J.I., Park J., Kim K.S. (2020). Fast and accurate desnowing algorithm for LiDAR point clouds. IEEE Access.

[33] Heinzler R., Piewak F., Schindler P., Stork W. (2020). Cnn-based lidar point cloud de-noising in adverse weather. IEEE Robot. Autom. Lett..

[34] Charron N., Phillips S., Waslander S.L. De-noising of lidar point clouds corrupted by snowfall. Proceedings of the 2018 15th Conference on Computer and Robot Vision (CRV).

[35] Roriz R., Campos A., Pinto S., Gomes T. (2021). DIOR: A hardware-assisted weather denoising solution for LiDAR point clouds. IEEE Sens. J..

[36] CompuWeather (2022). The Important Difference between Wet Snow and Dry Snow. https://compuweather.com/the-important-difference-between-wet-snow-and-dry-snow/.

[37] Oceanic N., Adminitration A. (2022). What Is the Difference between a Winter Storm Watch, Warning, and Advisory?. https://www.weather.gov/ilx/wwa_social.

[38] Administration K.M. (2022). Criteria for Advisory/Warning Information. https://web.kma.go.kr/eng/weather/forecast/standardwarninginfo.jsp.

[39] Lane M. (2022). Severe Weather Planning Guidance for HSE Services. https://www.hse.ie/eng/services/list/3/emergencymanangement/severe-weather/severe-weather-planning-guidance-for-hse-services-2024.pdf.

